# Dynamic Surface Interactions Enable the Self-Assembly of Perfect Supramolecular Crystals

**DOI:** 10.1021/acsami.4c11813

**Published:** 2024-10-17

**Authors:** Cem Tekin, Vincenzo Caroprese, Maartje M. C. Bastings

**Affiliations:** Programmable Biomaterials Laboratory, Institute of Materials, School of Engineering, Ecole Polytechnique Fédérale Lausanne, 1015 Lausanne, Switzerland

**Keywords:** supramolecular crystals, surface-assisted self-assembly, DNA nanotechnology, crystalline order, ion valency

## Abstract

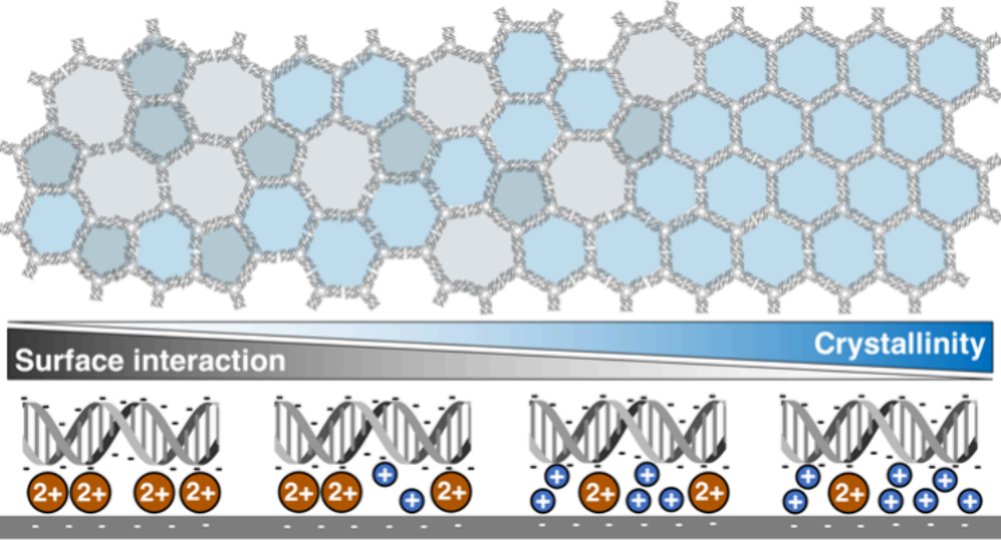

Supramolecular crystals arise from noncovalent interactions between macromonomers and allow for the engineering of dynamic functional materials. For two-dimensional (2D) crystals, the substrate surface can induce the formation of new polymorphs not available in solution, adding a layer of complexity to the supramolecular self-assembly process. Despite extensive studies on the 2D self-assembly of supramolecular crystals, unknowns remain regarding substrate–monomer interactions and the effects on network self-assembly and defect repair. Here, we used a DNA–mica model system to modulate and understand the impact of substrate–monomer interactions on the crystalline order. We controlled the surface interactions by tuning the Mg^2+^ concentration, varying the divalent cation type, and adjusting the relative concentration of divalent and monovalent cations. The competition between monovalent and divalent cations yielded nearly defect-free crystals with minimal polygon defects. These findings highlight the critical role of surface interactions in achieving high crystalline order, which is essential for optimizing the efficiency and performance of supramolecular functional nanomaterials.

## Introduction

Supramolecular two-dimensional (2D) crystals, which are surface-assisted assemblies of molecules held together by noncovalent interactions, represent an intriguing extension of traditional crystallography. The formation and morphology of these structures rely on the intermolecular forces, including hydrogen bonding, van der Waals interactions, and π–π stacking, alongside the additional complexity introduced by molecule–substrate interactions. Crystallization on surfaces opens new avenues in supramolecular chemistry with a potential for discovering crystal polymorphs not typically found through nucleation in solution.^1−3^ Consequently, surface-assisted supramolecular assemblies have received significant attention from the scientific community, with the aim of producing crystals with specific properties. These crystals hold promise for applications such as the development of advanced optoelectronic devices, sensors, and catalytic surfaces.^4,5^

Crystalline order is essential in 2D supramolecular crystals, as it directly influences their functionality in various applications. For instance, in hydrogen-bonded organic frameworks (HOFs), a well-defined crystalline structure ensures the stability and effectiveness in gas storage and separation.^6^ Similarly, in DNA origami sheets, precise nanoscale features are crucial for their potential roles in nanopatterning, where any disruption in the crystalline order could compromise their performance.^7^ Achieving such crystals requires building blocks with a certain rigidity, particularly at the interface, where interactions occur. *Interface flexibility*, which describes the relative positional and orientational freedom of attractive patches within a macromolecular binding unit, influences the efficiency and stability of these intermolecular interactions. We previously reported that while *interface flexibility* affects the size and shape of hexagonal networks formed by trimeric monomers, it has minimal or negligible impact on the crystalline order.^8^

Contrary to *interface flexibility*, the interaction between monomers and the surface was shown to impact the crystalline order in the self-assembly processes.^9−11^ Blunt-ended DNA 3-point-star (3PS) motif self-assembly into hexagonal networks provides a programmable platform to study the impact of these monomer–surface interactions on self-assembly.^12−15^ Commonly used substrates of DNA include mica^16−18^ and lipid bilayers,^14,19^ where electrostatic interactions govern DNA adhesion, making the valency of ions in the buffer crucial. Divalent cations can establish salt bridges between the negatively charged DNA and the negatively charged mica or lipid head,^18,20^ while monovalent cations compete with divalent cations for binding to both DNA and the substrate, hindering the formation of certain salt bridges.^18^ This interplay between divalent and monovalent cations is key for tuning the strength of DNA–surface interactions.

In this study, we interrogate the influence of monomer–surface interactions on the self-assembly of trimeric macromolecules. The objective of this work is to develop methodologies to enhance crystalline order in 2D supramolecular crystals using the DNA 3PS tile network as a model. Previous studies in literature highlight the important role of DNA–surface interactions in determining the order in close-packed^11,21,22^ and stacking-driven^21,23^ origami lattices. However, these existing examples mainly involve rigid DNA origami monomers, limiting defects to point defects or dislocations. Here, we conduct an in-depth analysis of the crystalline order in hexagonal lattices with numerous potential polygon defects. To explore the impact of the surface–DNA interaction, we employ Monte Carlo simulations of a patchy-particle model to guide the experimental strategies. By tuning the mica–DNA interaction strength through varying the concentration of Mg^2+^, replacing Mg^2+^ with other divalent cations, and introducing a monovalent cation (Na^+^) to compete with Mg^2+^, we significantly improved the crystalline order. We show that surface interactions dominate over the *interface flexibility* for network crystallinity. Finally, we attempted to self-sort two variants of 3PS with different arm lengths, allowing us to investigate the potential for selective assembly and sorting based on nuanced molecular variations.

## Results and Discussion

### Simulations Predict Highly Crystalline Networks for Weak Surface Interactions

Due to the directional nature of the π–π stacking interactions, arms of adjacent 3PS DNA tiles can form a π–π bond only when their helices parallelly align. We previously reported how flexibility at this interface effectively disrupts network growth, resulting in the formation of elongated islands with a few polygons. Contrary, rigid interfaces allow for the self-assembly of large polygonal networks.^8^ Although the macroscopic structural phenotype of the networks was heavily influenced by this *interface flexibility*, we did not record any significant relation toward crystalline order.^8^

**Figure 1:**
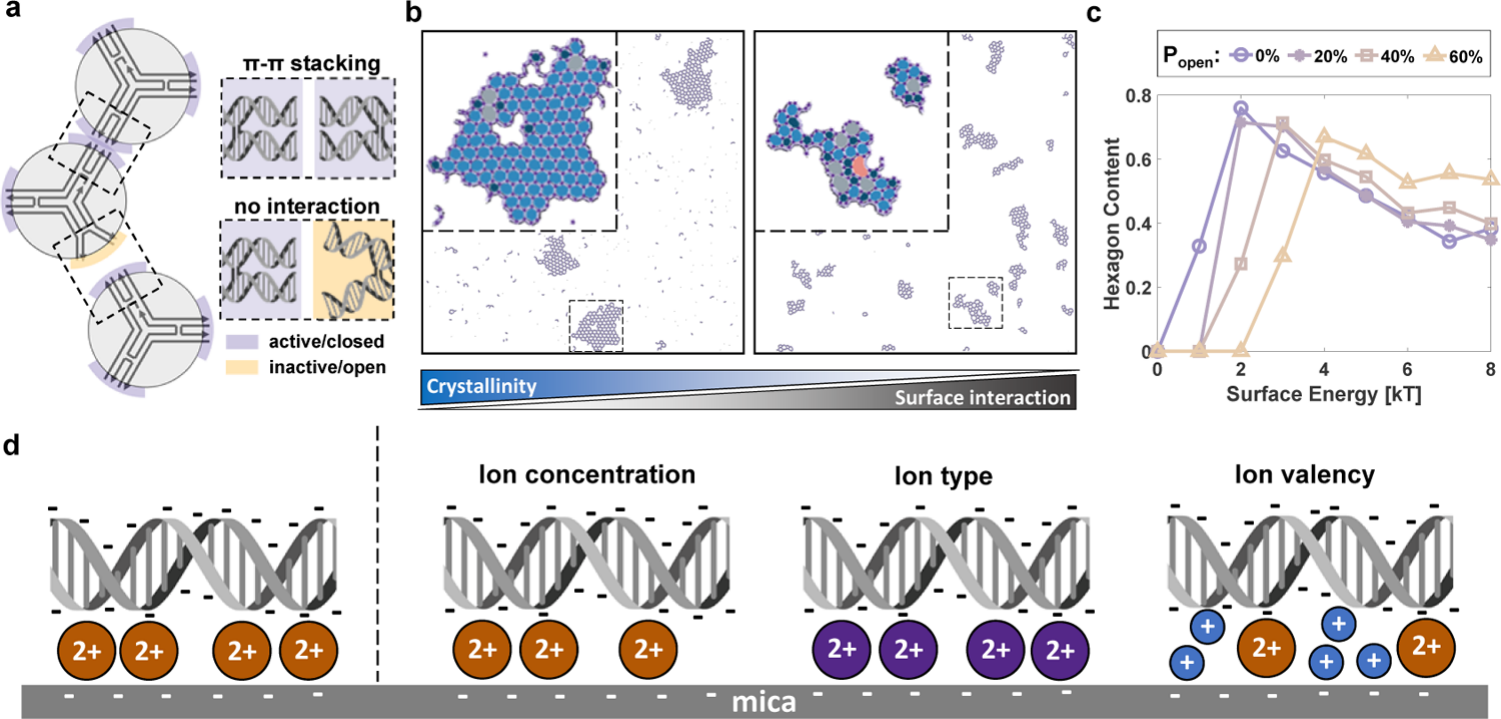
Impact of surface interaction energy. (a) Schematic representation of a patchy-particle model. If two active patches (purple) of adjacent particles overlap, the two particles attract each other. However, if one of the patches is inactive (yellow), mirroring the real-life scenario of DNA helices in an arm not being parallel, there is no interaction with the adjacent particle. Gray region is the repulsive core, preventing the overlap of particles. (b) Simulation frames where *P*_open_ is 0.2 and the surface interaction energies are 2 kT (left) and 4 kT (right). Zoomed-in region with polygon overlays is also presented. Dark blue, blue, gray, and pink represent pentagon, hexagon, heptagon, and octagon, respectively. As the surface interaction energy increases, we observe a decrease in the crystallinity and radiality. (c) Average hexagon content as a function of surface energy. Reported values are averages of 10 randomly selected frames after the convergence of simulations. Simulation parameters are σ = 1, δ = 0.038, θ_pw_ = 0.3, and ε = 6 kT. Parameters are selected to represent the π–π stacking interactions between 3PS tiles.^8^ (d) Three ways to tune DNA-mica interaction strength (from left to right): through tuning the concentration of the divalent cation, using a distinct divalent cation that interacts with mica and DNA differently, and adding monovalent cations to compete with the divalent cation. DNA helices are drawn using DNA helix drawer.^25^

Monomer–surface interaction strength has been shown to impact the crystallinity of surface-adhered lattices.^11,21,22^ Within the DNA model system, we investigated the impact of this parameter on the crystallinity of network assembly through Monte Carlo simulations using a patchy-particle model.^8^ This model was previously designed^8^ to reflect *interface flexibility* of the 3PS monomers, where each patch can be at active or inactive states, mirroring the closed (parallel dsDNA) and the open (unparallel dsDNA) configurations, respectively (Figure 1a). In this modified patchy-particle model, the monomer–surface interaction strength influences the frequency of patch state changes for bound particles. A stronger interaction with the surface makes it challenging for the patches of bound 3PS to break a bond or switch between the open and closed states. We varied this parameter, while simultaneously adjusting *interface flexibility* (probability of being at an open state, *P*_open_) to simulate the impact of surface interaction energy in 3PS systems. To analyze the simulation frames, an *in-house* developed detection algorithm was utilized (Methods). Crystalline order was quantitated through the polygon content and radiality through network density (ND).^24^ ND quantifies connectivity and organizational structure, adjusting for network size, and providing resilience against finite size effects. It is expressed as the ratio of observed number of polygons in a network to the number of polygons in the ideal honeycomb lattice formed by the same number of monomers.^8^ ND values near 1 indicate close-to-ideal radial networks, while decreasing ND suggests less organized, elongated assemblies.

We identified a trend as a function of surface interaction energies regardless of the *interface flexibility,* provided the interface is sufficiently rigid (*P*_open_ < 0.8). Initially, the monomers require 2–4 kT of surface interaction energy to form stable nuclei capable of growth; without this energy, they remain in a gaseous state (Figure S1). When sufficient surface interaction energy is provided, several nuclei formed in the early stages of the simulations develop into large crystalline networks (Figure 1b). Further increases in the surface interaction energies lower the overall crystalline order of these networks. This trend was confirmed by the quantitative analysis of hexagon content as a function of surface interaction energies, irrespective of *interface flexibility* (Figure 1c). For monomers with highly rigid interfaces (*P*_open_ ≤ 0.1), the hexagon content decreases by half as surface interaction energies increase from 2 to 8 kT. This decrease in hexagon content is also accompanied by a drop of ND from ∼0.9 to ∼0.7 (Figure S2). The less dynamic nature of monomer–monomer interactions, due to the increased energetic penalty of bond breaking at high surface interaction energies, leads to two outcomes. First, this increased bond stability results in the entrapment of polygon defects within the network, reducing the overall crystallinity. Second, the additional energetic penalty from surface interactions causes bonded particles’ patches to switch between open and closed states less frequently. Consequently, there is a higher likelihood of trapping particles with lower valencies (fewer than three active/closed patches), thereby reducing the radiality of the networks.^8^

Our in-silico insights reveal how variations in surface interaction energies impact crystalline order. To translate these insights into experimental conditions, we focus on modulating the surface interaction strength by tuning the density or stability of salt bridges between DNA and mica. To realize our goal of achieving highly crystalline networks, we followed three strategic approaches, as depicted in Figure 1d: (1) minimizing the density of salt bridges by tuning the Mg^2+^ concentration, (2) testing different divalent cations that are known to interact with DNA and mica differently than Mg^2+^, and (3) introducing monovalent cations to create competition with the divalent cations, thereby reducing the density of stable salt bridges.

**Figure 2:**
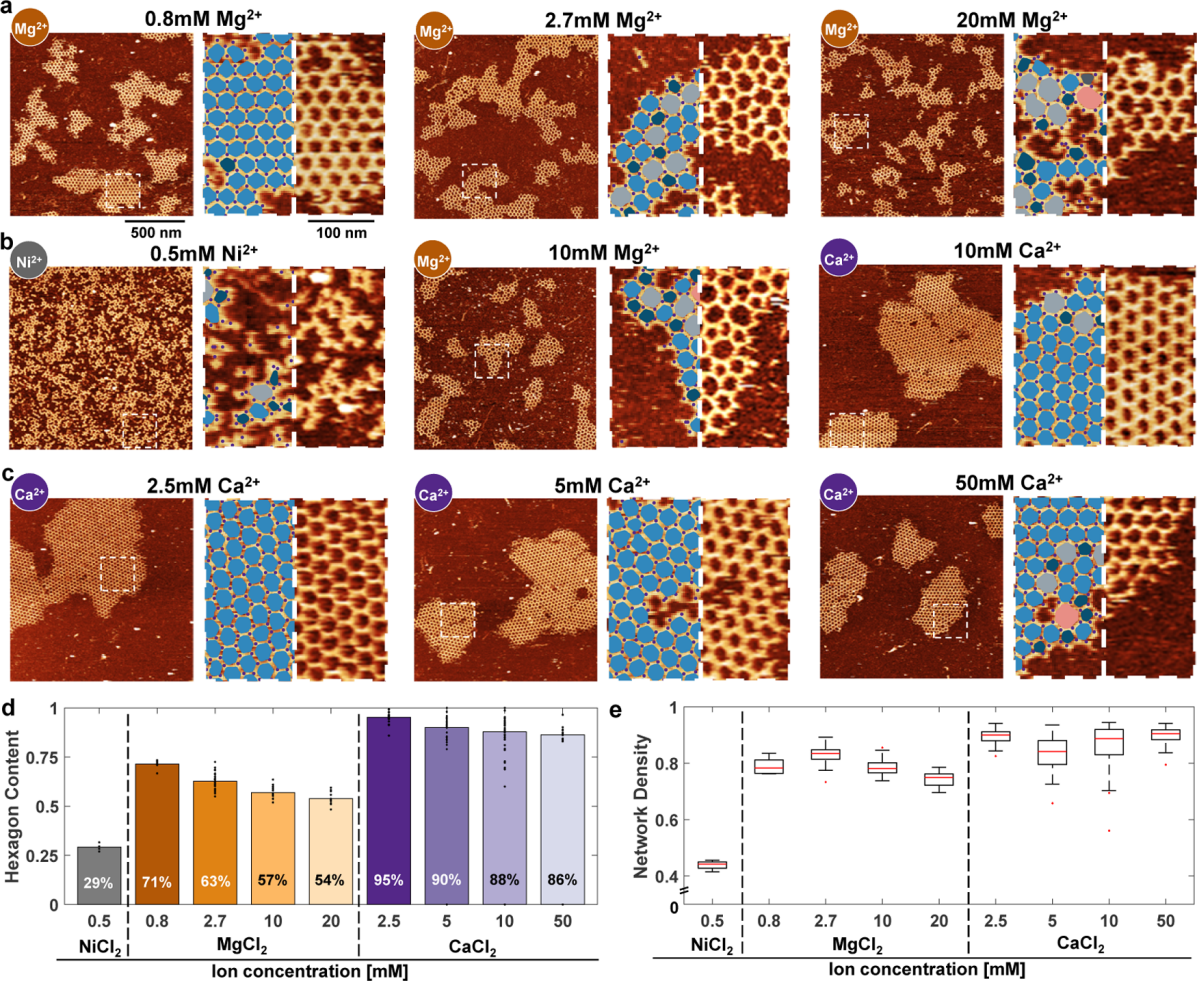
Effect of divalent cations. (a) AFM images of the steady-state networks formed at various Mg^2+^ concentrations. Minimum concentration of 0.8 mM was selected, as it was the lowest dilution that did not disrupt the integrity of individual monomers, while the upper limit of 20 mM was chosen once we observed that the properties of the networks reached a plateau, with no significant changes at higher concentrations. (b) AFM images of the steady-state networks formed in the presence of Ni^2+^, Mg^2+^, and Ca^2+^ (from left to right). (c) AFM images of the steady-state networks formed at various Ca^2+^ concentrations. Minimum Ca^2+^ concentration was set at 2.5 mM, as lower concentrations led to significantly reduced image quality due to decreased stability of the DNA monomers on the mica surface. Similar to Mg^2+^, the upper limit was determined by the point at which the network properties plateaued, ensuring that the full range of behavior was captured. (d) Weighted mean of the hexagon content as a function of the cation type and concentration. Each point represents the hexagon content recorded within a 1.5 μm × 1.5 μm area. For each condition, at least 8 images were analyzed. (e) Weighted mean of the network density as a function of cation type and concentration. For each condition, at least 8 images with an area of 1.5 μm × 1.5 μm were analyzed. Red lines indicate the median. Outliers, defined as data points beyond 1.5 times the interquartile range, are represented as individual points. For each ion type and ion concentration at a–c (on the left), an area of 1.5 μm × 1.5 μm and (on the right) higher magnification and overlay of the polygon identification algorithm (pentagons, dark blue; hexagons, blue; heptagons, light gray; octagons, pink) are shown. z-range was adjusted slightly for optimal data presentation.

The simplest strategy to reduce the density of salt bridges between DNA and mica is to lower the concentration of the divalent cation present in the buffer. Since the standard cation used in the imaging of DNA on mica is Mg^2+^, we first tested a concentration range from 0.8 to 20 mM (Figures 2a and S3). Following overnight assembly, the blunt-ended 3PS system showed a remarkable shift in order: with decreasing Mg^2+^ concentration, the crystallinity improved visibly. This expansion of crystalline domains directly correlates with the diminishing surface interaction strength suggesting the validity of the simulations of the patchy-particle model. Subsequently, we explored the effect of Ni^2+^ and Ca^2+^ ions on the 3PS system (Figure 2b). Ni^2+^, a transition metal ion, exhibits a strong affinity for DNA, particularly with the ring site of guanine and adenine, and the exocyclic sites of guanine.^26^ This strong binding is attributed to the unfilled d orbitals of Ni^2+^, allowing it to form covalent complexes with DNA bases, as demonstrated by Raman spectroscopy.^26^ Due to the high ionic potential of Ni^2+^ ions, they can also bind to mica stronger than the alkaline earth metal cations.^18,27−29^ As a result, Ni^2+^ ions, even at 0.5 mM (higher concentrations resulted in the formation of visible salt aggregates), unsurprisingly halted the formation of crystals, keeping the system in the diffusion limited aggregates (DLA) observed in the simulation of the patchy-particle model at high surface interaction strength (Figure S1). On the contrary, the introduction of Ca^2+^ ions facilitated the formation of nearly perfect crystals (Figures 2c and S4). Across all tested Ca^2+^ concentrations, the resulting lattices exhibited greater radial symmetry and higher crystalline order compared to those formed with Mg^2+^. The analysis employing our particle detection algorithm revealed a direct correlation between crystalline order and the concentration of the divalent cation (from 54 to 71% for Mg^2+^ and 86 to 95% for Ca^2+^, Figure 2d), while radiality exhibited no discernible correlation with ion concentration (Figure 2e).

This significant difference between Mg^2+^ and Ca^2+^ presents a complex and intriguing phenomenon. Unlike Ni^2+^, which interacts through nucleobases, Mg^2+^ and Ca^2+^ ions primarily interact with the phosphate groups of nucleic acids rather than the bases.^26,30−34^ However, the nature of these interactions differs due to the distinct properties of Mg^2+^ and Ca^2+^. Although Ca^2+^ is more electropositive, Mg^2+^ exhibits a higher affinity for phosphate groups because of its smaller ionic radius and higher charge density.^30,31^ This higher charge density leads to greater polarization of the phosphate oxygen atoms, resulting in a stronger and more stable coordination. The interaction between Mg^2+^ and the phosphate group is generally more thermodynamically stable due to the smaller ionic radius and higher charge density of Mg^2+^, which allows it to form stronger, more localized interactions with the phosphate groups.^35^ In contrast, Ca^2+^, with its lower charge density, forms less rigid complexes, which increases the conformational flexibility of the DNA structure on mica.^35^ This flexibility could enable Ca^2+^-bound DNA monomers to rearrange more easily on the mica surface, enhancing the crystallinity of the networks without significantly disrupting the salt bridges.

### Monovalent vs Divalent Cation Competition Yields Defect-Free Crystals

**Figure 3:**
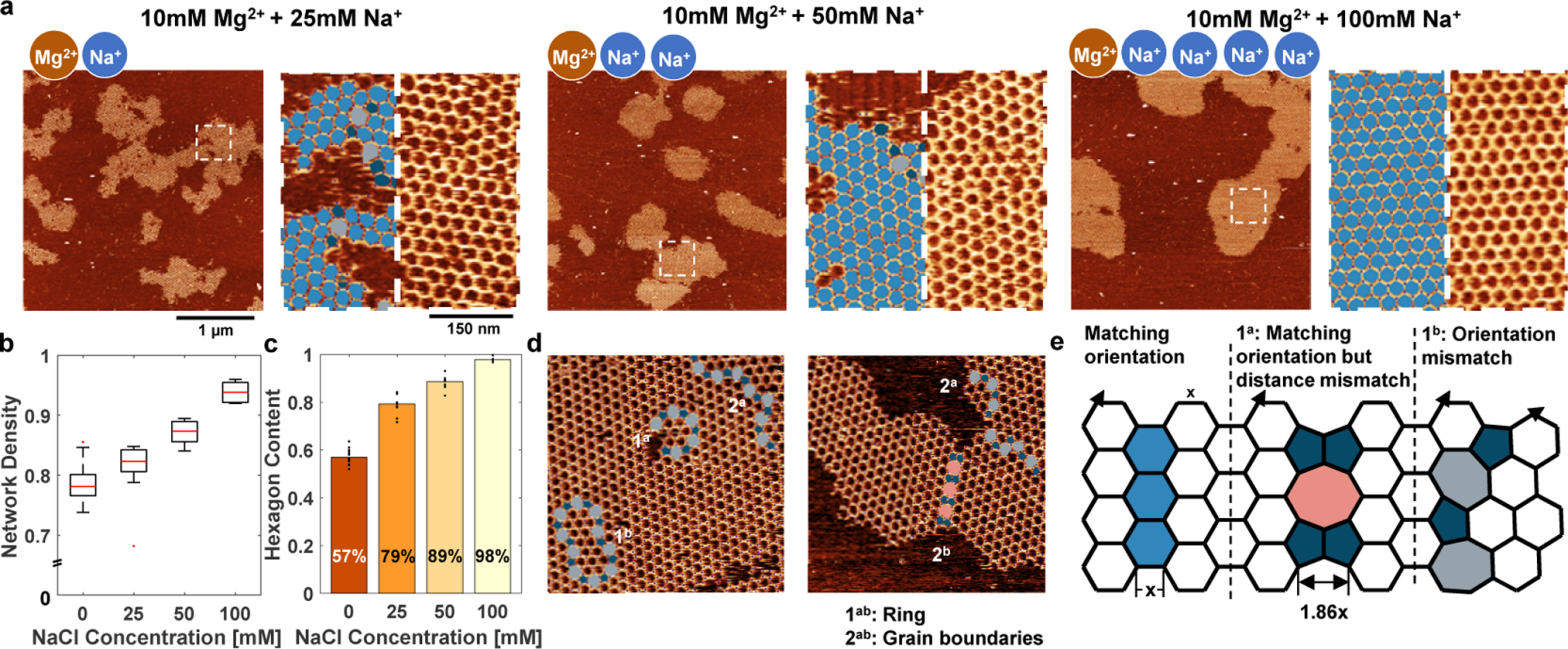
Tuning the DNA-mica interaction through ion valency. (a) AFM images of the steady-state networks formed at different Na^+^ concentrations. Na^+^ concentrations were tested up to 100 mM, as higher concentrations led to a significant reduction in DNA monomer adsorption on the mica surface, indicating less favorable conditions for adsorption. For each concentration (on the left), an area of 3 μm × 3 μm and (on the right) higher magnification and overlay of the polygon identification algorithm (pentagons, dark blue; hexagons, blue; heptagons, light gray; octagons, pink). (b) Weighted mean of the network density as a function of Na^+^ concentration. For each condition, at least 12 images with an area of 1.5 × 1.5 μm were analyzed. Red lines indicate the median. Outliers, defined as data points beyond 1.5 times the interquartile range, are represented as individual points. (c) Weighted mean of the hexagon content as a function of the Na^+^ concentration. Each point represents the hexagon content recorded within a 1.5 μm × 1.5 μm area. For each condition, at least 12 images were analyzed. (d) Typical defects that we observed in the steady-state networks (highlighted on AFM images). Formation of ring defects is discussed further in Figure S6. (e) Schematic representation of different grain boundaries. If two grains align perfectly, then the grains can emerge without formation of any defects. If the crystal orientation is the same for two grains but the gap between them is not matching, then a grain boundary with a repeating unit of two pentagons and an octagon forms. However, because the grains do not have the same orientation, they tend to form a grain boundary with a repeating unit of a pentagon and a heptagon. z-range was adjusted slightly for optimal data presentation.

Monovalent cations, specifically Na^+^, compete with Mg^2+^ and effectively reduce the number of salt bridges between 3PS monomers and the surface.^18^ We exposed 3PS to 10 mM Mg^2+^ and varying Na^+^ concentrations from 0 to 100 mM and imaged the resulting lattices following overnight incubation on mica (Figure 3a). As the concentration of Na^+^ increases, the islands become larger and more radial (Figure 3b) and the hexagon content rises from 57 to 98% (Figure 3c). The addition of Na^+^ reduces the number of salt bridges, allowing the monomers to diffuse more freely, which decreases their likelihood of being trapped on the surface. However, unlike simply reducing the Mg^2+^ concentration, which also lowers the overall number of salt bridges, Na^+^ introduces a dynamic process where salt bridge formation and dissociation occur more frequently. Mg^2+^, as a divalent cation, forms direct and stabilizing salt bridges between DNA and the surface, effectively anchoring the monomers. In contrast, monovalent Na^+^ shields the negative charges on DNA and the surface rather than forming such stabilizing interactions. This difference likely leads to more transient salt bridges in the presence of Na^+^, increasing the monomer mobility. This increased mobility facilitates the formation of larger, more ordered hexagonal domains. The monomers can frequently adjust their positions, preventing them from becoming too rigidly anchored to the surface and enabling them to find optimal arrangements more easily.

Upon careful examination of the AFM images, we identified recurring defect types (Figures 3d and S5–S8). Two distinctive defect categories emerged: (1) ring defects, circular defect patterns that are composed of equal number of pentagons and heptagons, and (2) grain boundaries. Ring defects manifested usually under conditions of heightened concentration, while 3PS monomers densely populate the surface (Figure S5). We have never recorded the correction of it even some vacancy defects within the island migrates through it, showcasing their local stability. At the intersection of two growing grains, a grain boundary forms unless the lattice orientation and the relative distance of the two grains perfectly match (Figure 3e). In cases where they do not align, grain boundaries emerge in two distinct patterns: one composed of a repeating unit with a pentagon and a heptagon and the other featuring an octagon and two pentagons as the repeating unit. The first scenario occurs when there is again no phase between the lattice orientation of the two growing grains, but the relative distances do not match. Unable to move, the lattices compensate by forming an octagon and two pentagons between the two grains. The second case, involving a phase angle between the grains, leads to the formation of grain boundaries, comprising pentagons and heptagons. In addition to these grain boundaries, heptagons were consistently neighbored by two pentagons, while octagons were flanked by four pentagons (Figure S8). This structural adjustment corrected for the angles altered by larger defects in the lattice.

### Ion Competition (Monovalent vs Divalent) Improves the Crystalline Order Regardless of the *Interface Flexibility*

**Figure 4:**
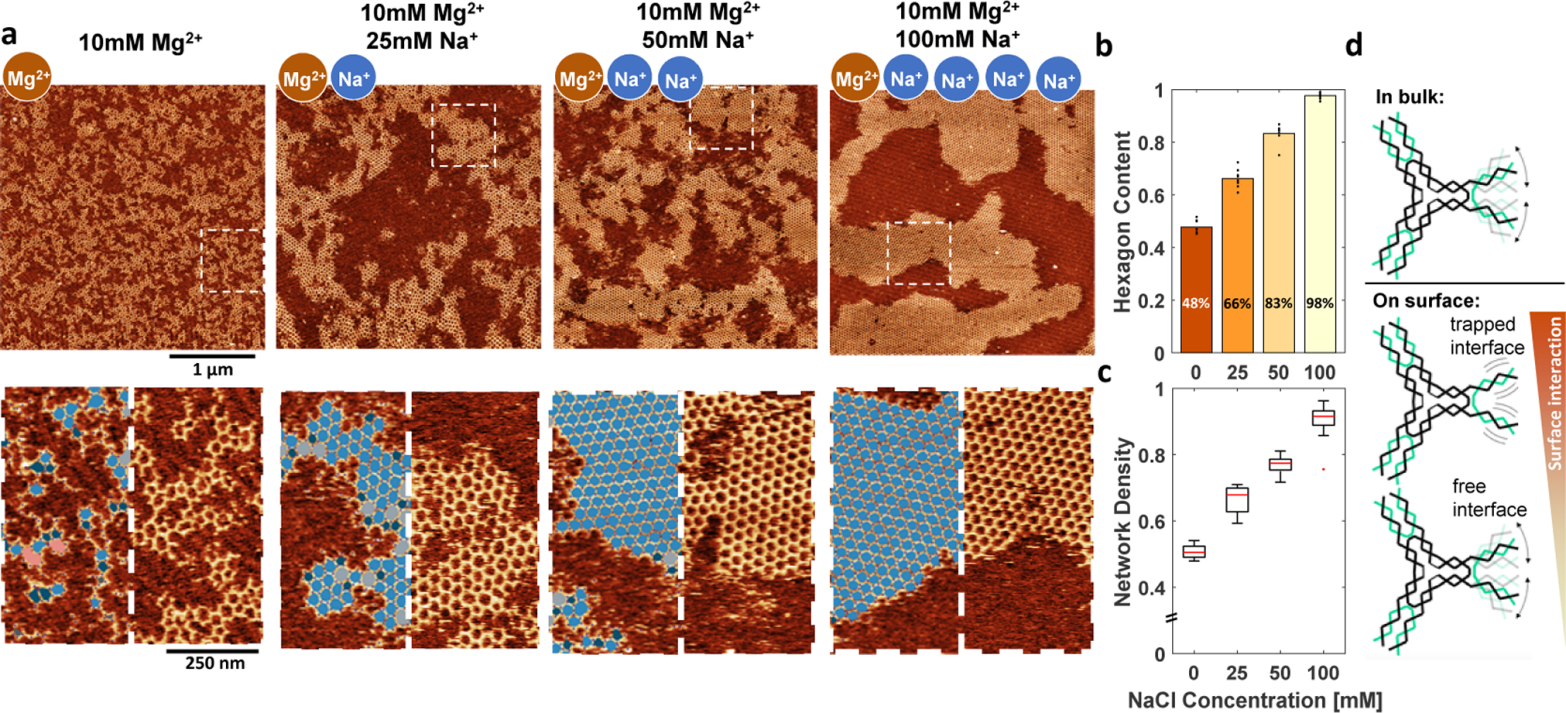
Influence of the interface mobility. (a) AFM images of the steady-state networks formed at 10 mM Mg^2+^ and various Na^+^ concentrations. For each concentration (on top), an area of 3 μm × 3 μm and (bottom) higher magnification and overlay of the polygon identification algorithm (pentagons, dark blue; hexagons, blue; heptagons, light gray; octagons, pink). *z*-range was adjusted slightly for optimal data presentation. (b) Weighted mean of the hexagon content as a function of the Na^+^ concentration. Each point represents the hexagon content recorded within a 1.5 μm × 1.5 μm area. For each condition, at least 8 images were analyzed. (c) Weighted mean of the network density as a function of Na^+^ concentration. For each condition, at least 8 images with an area of 1.5 μm × 1.5 μm were analyzed. Red lines indicate the median. Outliers, defined as data points beyond 1.5 times the interquartile range, are represented as individual points. (d) In bulk solution, 3PS interfaces are highly mobile and can assume many conformations. When the degree of freedom is lowered due to adsorption on a surface, the mobility of this interface is heavily dependent on the strength of the interaction with the surface. If the interaction is strong, then the interface may be trapped in inactive/open states. On the other hand, if the interaction is weak, the high mobility of the interface may make the interface flexibility less important since the speed of interface movement may surpass the speed of 3PS diffusion.

The *interface flexibility* was shown to be essential in determining the assembly mechanism that defines the morphology of networks.^8^ A 3PS monomer with higher *interface flexibility* (each arm is extended by half a turn, Figure S9) was shown to lack radial growth in the presence of only Mg^2+^, resulting in elongated networks with few polygons.^8^ Monte Carlo simulations of the patchy-particle model presented in Figure 1 suggest that reducing the surface interaction strength improves not only the crystalline order but also the radiality regardless of *interface flexibility* (Figure 1c). Therefore, we experimentally studied the self-assembly of this 3PS monomer with a higher *interface flexibility* in a less restrictive environment. By allowing the 3PS arms to diffuse more freely on the surface in the presence of 10 mM Mg^2+^ and varying concentration on Na^+^, we aimed to recover the radial growth while also improving the crystalline order (Figure 4). AFM imaging after an overnight incubation yielded notable results (Figure 4a): with increasing Na^+^ concentration (e.g., reducing surface interaction strength), the 3PS with a flexible interface converges to the behavior of the standard (rigid interface) 3PS. We observed not only an increase in the hexagon content from 48% up to the level of an almost defect-free crystal, 98% (Figure 4b), but also the recovery of the radial island growth (ND increased from 0.52 to 0.92, Figure 4c) with a reduction in the surface interaction, confirming the findings of simulations.

In bulk solution, the interface of a 3PS is quite flexible, allowing various conformations (Figure 4d). However, upon deposition onto a surface, this flexibility may diminish, depending on the strength of the interaction with the surface. Under conditions of high surface interaction strength, such as in the presence of Mg^2+^ or Ni^2+^, the interface may become trapped in an inactive/open state, particularly if the interface is flexible. Therefore, a 3PS with a flexible interface is likely to have an increasing number of interfaces trapped in an inactive/open state, resulting in a reduction in the effective valency and, thus, the formation of elongated islands instead of radial ones. However, as the Na^+^ concentration in the buffer increases, disruption of Mg^2+^-mediated salt bridges reduces surface interaction strength, allowing the interface more freedom to move. As interface mobility increases, the effects of *interface flexibility* diminish because the speed of interface movement exceeds that of particle diffusion. Consequently, the behavior of 3PS with a flexible interface converges to that of the standard 3PS.

### Homogeneous Geometries Create Mixed Crystals but Prevent Self-Sorting

**Figure 5:**
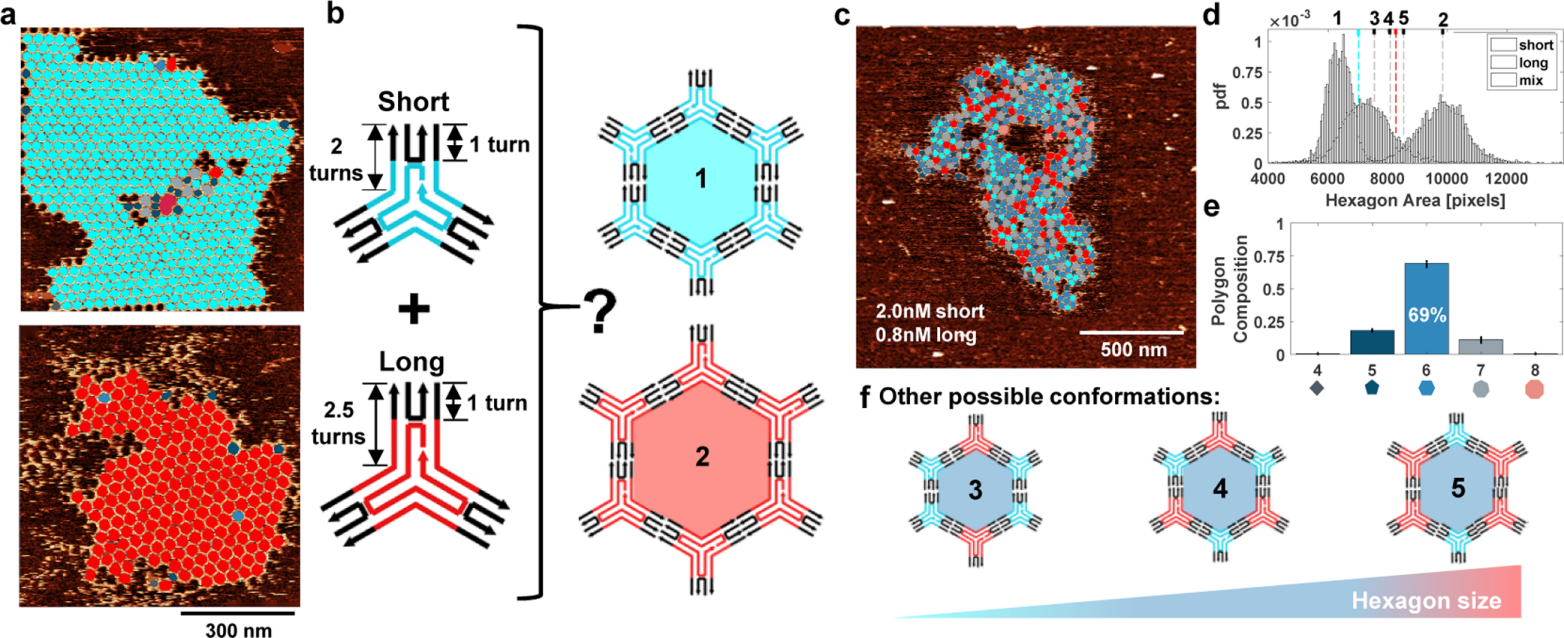
Pseudocrystalline networks of short and long 3PS. (a) AFM images of the steady-state networks formed by short (top) and long (bottom) 3PS. Hexagons with areas smaller than the lowest threshold (95th percentile of the hexagon area distribution formed by short 3PS) were classified as hexagons formed by short 3PS and painted with cyan. Hexagons with areas bigger than the highest threshold (5th percentile of the hexagon area distribution formed by long 3PS) are classified as hexagons formed by long rigid 3PS and painted with red. Rest of the hexagons with moderate areas were painted with blue. (b) Schematic representation of a self-sorting experiment. (c) Steady-state network formed by short (2.0 nM) and long rigid (0.8 nM) 3PS. (d) Probability density functions (pdf) of hexagon areas for short only (cyan), long rigid only (red), and self-sorting experiment (blue). (e) Distribution of the polygons formed at the steady-state networks at the self-sorting experiment. Each point represents the specific polygon content recorded within a 1.5 μm × 1.5 μm area. At least 12 images were analyzed. (f) Other possible conformations of hexagons that are formed by short and long 3PS that would induce only minimal stress in a lattice.

With multiple 3PS monomers of various sizes in hand, we aimed to test whether a dimensional mismatch is sufficient to drive self-sorting. Self-sorting is a common phenomenon in supramolecular systems, where several species in a complex mixture exhibit distinct affinities toward each other.^36−38^ Mao and colleagues extended this concept to geometry-based self-sorting using DNA 3PS and 4PS with the same local affinity but a global geometric mismatch.^39^ The internal stress induced on the lattices prevented the formation of mixed species lattices as multiple species could not be accommodated efficiently (preventing the maximization of local interactions). A similar outcome could occur if two species with different sizes are mixed, which we explored by mixing a long (Figure S10) and a short 3PS, which only differ in arm length (with identical local affinity and *interface flexibility*, Figure 5a,b) at various concentrations in a solution containing 10 mM Mg^2+^ and 100 mM Na^+^ (Figure 5c). To identify the types of 3PS present in each hexagon observed, we analyzed the area occupied by hexagons in the acquired AFM images (Figure 5d). Self-sorted lattices are expected to yield two separate normal distributions of hexagon sizes, with one corresponding to hexagons formed by short 3PS (cyan) and the other associated with long 3PS (red). Hexagon size analysis revealed a broad distribution (blue) with a single peak between short and long 3PS hexagon size distributions, indicating a lack of noticeable self-sorting. Interestingly, the resulting lattices exhibited more defects compared to both short-only and long-only configurations, likely due to the increased complexity in assembling mixed species, which can lead to misalignments and irregularities that are less common in uniform assemblies (Figure 5e). Similar experiments at different 3PS concentrations (Figures S11–S14) and using the short 3PS and long 3PS with flexible interface yielded almost identical results (Figure S15).

We propose that the absence of self-sorting can be attributed to the well-matched geometries of short and long 3PS, as illustrated in possible configurations in Figure 5f. In these configurations with various spanned areas, the 3PS monomers continue to maximize the number of local interactions without disrupting the global structure. Additionally, the dynamic nature of the assembly allows individual 3PS to interact with already established networks one by one, facilitating sequential movement to form a compatible hexagon with an optimal area that avoids stressing the global structure. A remarkable heterogeneous but hexagonally ordered pseudocrystal is the result.

## Conclusions

In this study, we explored the experimental landscape to self-assemble defect-free crystalline supramolecular networks with DNA 3PS monomers. This established system assembles into perfect crystals when strong affinity sticky-ends interactions were applied.^12^ With blunt-ended 3PS, the interface interactions are of much lower affinity, and previous work presented *interface flexibility* as dominating parameter for network self-assembly, but not for crystalline order.^8^ Here, we predominantly focused on the precise modulation of surface–monomer interactions. Interaction with the surface is a crucial aspect of such an assembly, with important implications especially for the crystalline order. Employing simulations of patchy-particles as a guide, we unveiled both the crystalline order and the radiality of the islands improving as the surface–monomer interaction weakens.

Translating insights from simulations into experiments, we systematically varied the monomer–surface interaction strength through three distinct methodologies. First, manipulating the Mg^2+^ concentration yielded a noteworthy enhancement in crystallinity as salt bridge density diminished at lower Mg^2+^ concentrations. Second, experiments featuring divalent cations Ni^2+^ and Ca^2+^ demonstrated a distinct contrast: the former impeded crystal formation due to strong interactions with DNA and mica, while the latter, potentially owing to its lesser interaction strength with mica, fostered increased DNA monomer mobility, resulting in nearly defect-free crystals (95% crystallinity). The most remarkable outcomes originated from the third method, introducing a competitive dynamic between a divalent cation (Mg^2+^) and a monovalent cation (Na^+^). At 10 mM Mg^2+^ and 100 mM Na^+^, micrometer-sized lattices boasting an outstanding 98% crystallinity were achieved, underscoring the profound impact of controlled monomer–surface interactions. We observed the consistent occurrence of specific defect types, where a pentagon or an octagon was consistently neighbored by two or four pentagons, respectively, to uphold the integrity of the global lattice structure.

Experiments conducted with 3PS featuring a flexible interface demonstrated that large crystals could be formed by weakening the interaction with the surface. This outcome appears to be counterintuitive as *interface flexibility* was previously shown to negatively affect the nucleation of networks. This disruption is the result of a reduction of effective valency of the 3PS due to structural flexibility at the binding interface. For strong surface interactions, this effective valency is locked in place, preventing all arms to partake in lattice formation. However, modification of the surface interactions opens the possibility for flexible interfaces to continuously rearrange. The rate at which a surface-adsorbed particle transitions between states is controlled by a kinetic barrier related to local surface detachment. When this rate is sufficiently higher than that of particle diffusion, particles in close proximity can consistently achieve available states. Consequently, particles will always appear to have constant valency (3 in our case), and large networks can indeed grow. We argue that the modifications performed in this study achieve this balance, causing both standard and flexible 3PS to converge and form a perfect radial crystal at 10 mM Mg^2+^ and 100 mM Na^+^.

Finally, we explored if a conserved molecular geometry but difference in size would be enough to drive self-sorting. Unlike traditional self-sorting in natural supramolecular systems, which relies heavily on species with distinct affinities, our approach involved species with identical local affinities but varying sizes that could potentially affect the global lattice structure. Nevertheless, the high compatibility between short and long 3PS effectively mitigated the stress required for successful self-sorting resulting in the assembly of a mixed-sized hexagonal pseudocrystal.

Our current exploration into DNA 3PS self-assembly has provided valuable insights into attaining defect-free crystals by controlling surface interactions. These findings not only deepen our understanding of the fundamental physics governing nanoscale material behavior but also demonstrate practical improvements in crystalline order. By controlling surface interactions, we can better tailor the properties of 2D crystals, potentially enhancing the design and fabrication of dynamic functional materials. Such advancements can lead to more efficient and reliable nanodevices and sensors, improving the performance of various nanoscale applications.

## Methods

### Patchy-Particle Simulations

The simulation engine developed from the work of Hedges^40^ can be found in https://github.com/mosayebi/PatchyDisc.^41^ The patchy-particle model used in the simulations was described elsewhere.^8^ 1500 particles with inactive patches, random positions, and random orientations were initialized on a 2D area of 150 × 150 normalized unit,^2^ which represents and area of 1.6 × 1.6 μm^2^, to match the densities measured in our AFM images. Each simulation was run for 2.4e8, where we always observed convergence. The interaction limit is set at 0.038, which corresponds to ∼0.6 nm. The patch width (θ_pw_) was set to 0.3, and the interaction energy (ε) was set to 6 kT. Recorded trajectories were transformed into visual representations through a Python script and VMD. Frame analysis was carried out in MATLAB, employing methodologies akin to those applied in processing the experimental AFM data, excluding the segmentation.

### DNA 3PS Preparation

The details about individual 3PS preparation are explained elsewhere.^8^ Briefly, for each 3PS motif, a solution with 0.6 μM S1x, 1.8 μM S2x, and 1.8 μM S3x (bought from IDT Inc., sequences listed in Table S1) and 5 mM TRIS (Bio-Rad), 1 mM EDTA (ITW Reagents), and 10 mM MgCl_2_ (Sigma) or CaCl_2_ (Sigma) at pH 8.0 was prepared and annealed by slowly cooling from 80 °C to room temperature in 4 h. The product was analyzed by Native PAGE (6%) to check if there is some not folded excess ssDNA in the solution. In the case of excess strands in the product, the annealing solution was loaded on a 3% agarose (Sigma) gel and ran at 60 V in 0.5x TBE (Thermo Scientific) and 10 mM MgCl_2_ for 150 min in an ice-cooled water bath. The 3PS band was excised, and it was extracted from the gel by centrifugation in a Freeze ‘N Squeeze gel extraction spin column (Bio-Rad) for 20 min at 3000 RCF, 4 °C. The buffer was replaced by the annealing buffer using a Vivaspin 500, MWCO 3000 (Sartorius), following manufacturer’s instructions (except centrifugation at maximum 3000 RCF). The purified sample was characterized by Native PAGE shown elsewhere.^8^

### AFM Imaging

The mica (grade V1, Ted Pella) was incubated with 40 μL of 3PS solution at 3–12 nM and the desired buffer conditions. The stock solution containing ∼0.3 μM DNA 3PS was diluted as follows:

- For experiments with Mg^2+^ and Ca^2+^, the stock solution was diluted with Milli-Q water or the corresponding Mg^2+^/Ca^2+^ buffer solutions (pH 8.0) at the desired concentrations. The lowest Mg^2+^ concentration (0.8 nM) was chosen to ensure the integrity of the monomers, while Ca^2+^ concentrations started from 2.5 mM to avoid significant reductions in image quality, likely due to decreased DNA monomer stability on mica. The upper limits were defined based on when further increases in concentration no longer affected the network properties.
- For experiments with Ni^2+^, the stock solution was diluted with Milli-Q water, with a small amount of Ni^2+^ buffer added to achieve the desired concentration. To prevent issues with salt aggregate formation, Ni^2+^ was kept at a low concentration.
- For experiments with Na^+^ and Mg^2+^, the stock solution was diluted using a buffer containing 10 mM MgCl_2_ and varying amounts of NaCl to reach the desired final Na^+^ concentration (pH 8.0). Concentrations above 100 mM Na^+^ were avoided, as they significantly reduced DNA monomer adsorption on the mica surface, indicating unfavorable conditions for adsorption.

After an overnight incubation, the resulting lattices were imaged in tapping mode in liquid on a Cypher VRS (Asylum Research Inc.) using a BioLever mini cantilever (BL-AC40TS-C2, Olympus). Pixel resolution for AFM images was set according to the scanning area, while maintaining a pixel size of ∼3 nm.

### Image Processing and Analysis

The simulation frames and AFM images were processed and analyzed by a custom MATLAB script. Each AFM image is capped between 0.5 and 99.99 percentile (height) to remove possible outliers. Then, a polynomial line removal excluding the foreground (determined by Otsu’s thresholding) was applied twice followed by capping the image between 0 (determined by the median of the background) and maximum allowed height determined by a dynamic thresholding function. The dynamic thresholding function was implemented to address significant height variations between different imaging lines. This was deemed necessary as height fluctuations led to misidentification of foreground and background pixels, necessitating a more stringent capping strategy when a higher proportion of background pixels was present. The rest of the processing steps (segmentation and skeletonization, polygon and particle detection, and collection of observables per connected component) are detailed elsewhere.^8^

## Supporting Information Available

Additional figures and full list of DNA sequences (PDF)
