# Supplementary material for: Dynamic Surface Interactions Enable the Self-Assembly of Perfect Supramolecular Crystals
Source: ACS Appl Mater Interfaces. 2024 Oct 17;16(43):59040–8. doi: 10.1021/acsami.4c11813 (PMC11533158; doi:10.1021/acsami.4c11813)
Supplement: Supplementary file 1 — am4c11813_si_001.pdf [file am4c11813_si_001.pdf]

Supporting Information for

Dynamic surface interactions enable the self-assembly of perfect supramolecular crystals

Cem Tekin[1], Vincenzo Caroprese[1], Maartje M.C. Bastings[,1]*

1 Programmable Biomaterials Laboratory, Institute of Materials, School of Engineering, Ecole Polytechnique Fédérale Lausanne, Lausanne, 1015, Switzerland

* Corresponding author: maartje.bastings@epfl.ch

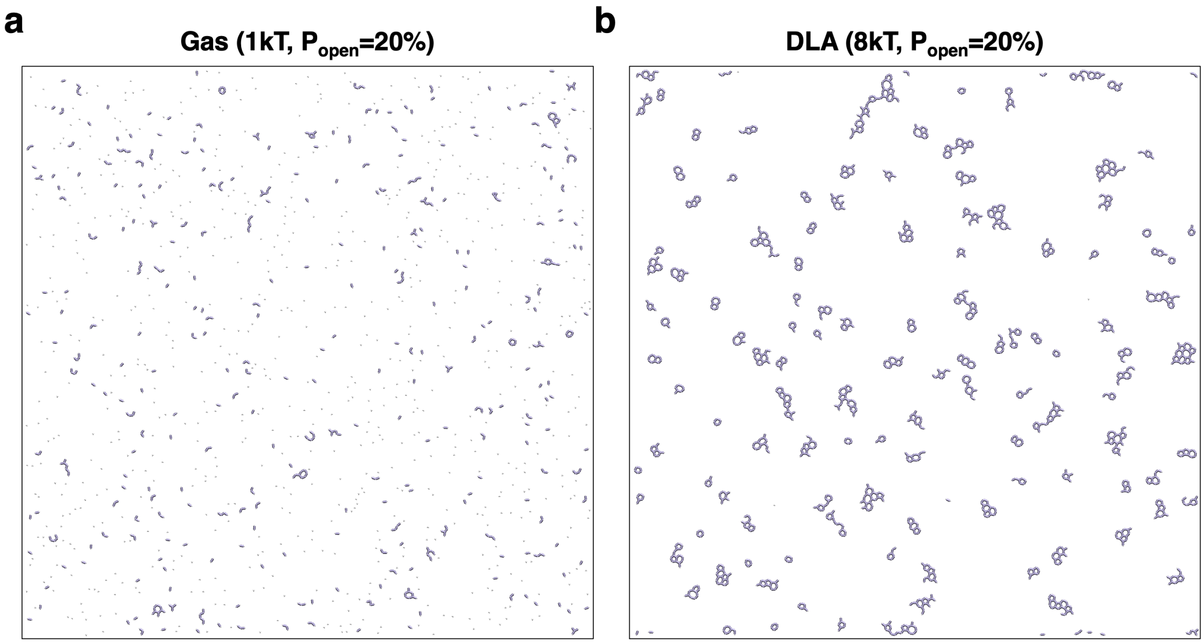

Figure S1: Representative frames from the Monte Carlo simulations of the patchy-partice model.
a, Gaseous state observed at 1kT surface interaction energy and P_{open} of 0.2. This state occurs when interactions are too weak to form stable islands, typically at very weak surface interaction energies or high interface flexibility (P_{open}). **b,** Diffusion-limited aggregate (DLA) state observed at 8kT surface interaction energy and P_{open} of 0.2. This state occurs when interactions are too strong, making established bonds impossible to break, typically at very strong surface interaction energies.

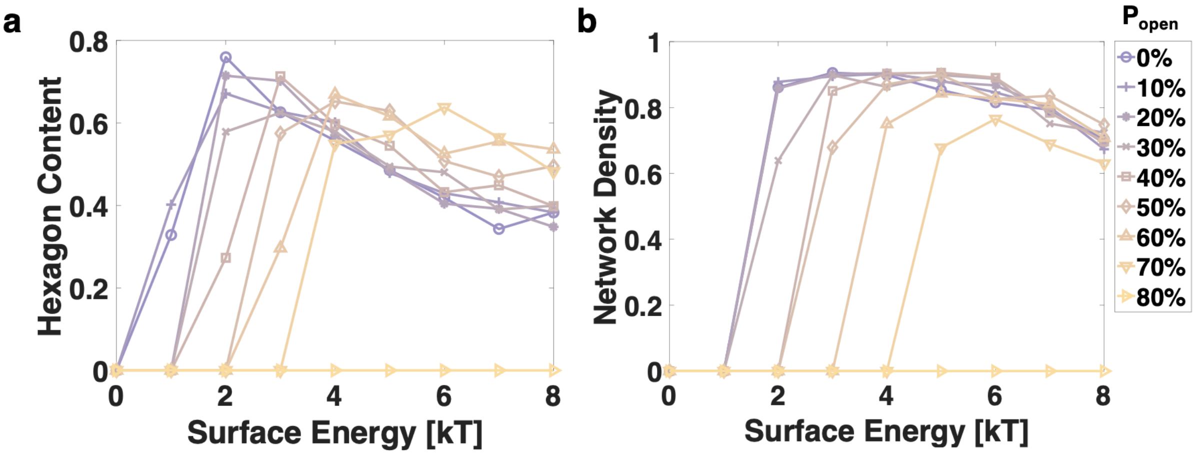

Figure S2: The analysis of the Monte Carlo simulations of the patchy-partice model.
a, Average hexagon content as a function of surface energy. Additional datapoints are added which were not used in Fig. 1 due to limitations of space. **b,** Weighted average Network Density (ND) as a function of surface energy. ND in this context is a measure of radiality. It indicates how close an island is to a perfectly radial island (1 indicates a perfectly radial island). The analysis was conducted considering islands bigger than 23 particles. If there are not any islands bigger than 23 particles, an ND value of 0 is assigned.

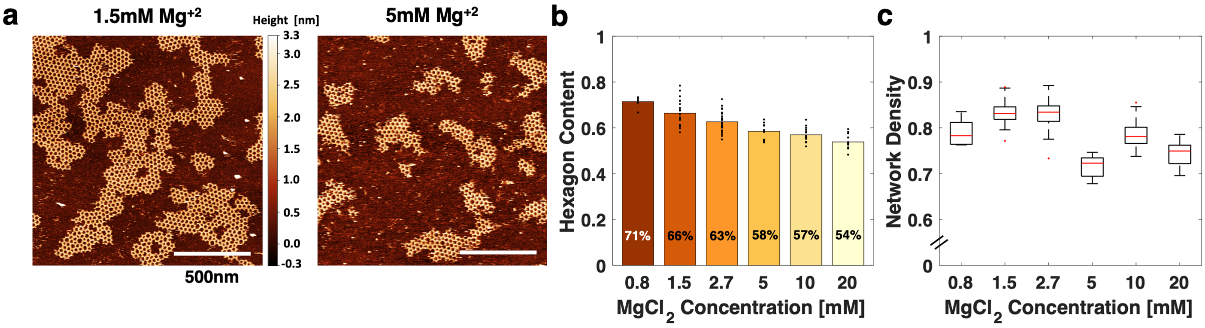

Figure S3: Steady-state networks of short 3PS in the presence of Mg^{2+}.
This figure includes the data from Fig. 2, with the addition of two additional data points that were not presented in Fig. 2 due to limited space.
a. AFM images of the steady state networks in the presence of 1.5mM (left) and 5mM (right) $MgCl_2$. The colorbar for height is representative of all of the AFM images presented in this study. Each AFM image displays only the DNA 3PS motif, identifiable by its typical height of 2 nm. The contrast has been slightly modified for each AFM image to enhance the clarity of the data. **b.** Weighted mean of the hexagon content as a function of the $MgCl_2$ concentration. **c.** Weighted mean of the Network Density of the resulting networks as a function of the $MgCl_2$ concentration.

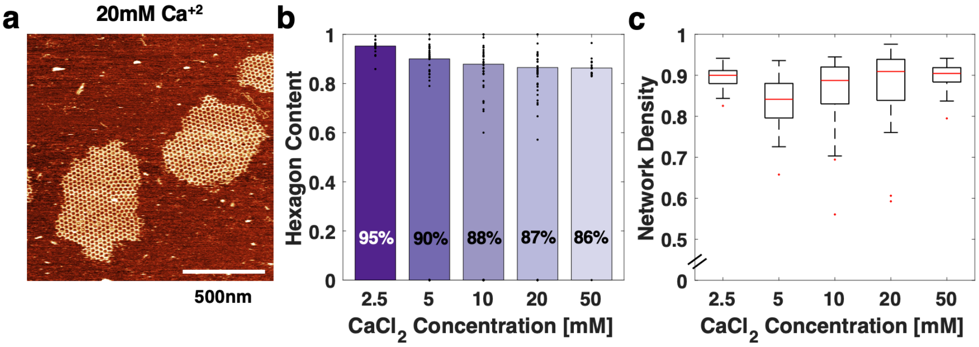

Figure S4: Steady-state networks of short 3PS in the presence of Ca^{2+}.
This figure includes the data from Fig. 2, with the addition of an additional data point that was not presented in Fig. 2 due to limited space.
a. An AFM image of the steady state networks in the presence of 20mM $CaCl_2$. **b.** Weighted mean of the hexagon content as a function of the $CaCl_2$ concentration. **c.** Weighted mean of the Network Density of the resulting networks as a function of the $CaCl_2$ concentration.

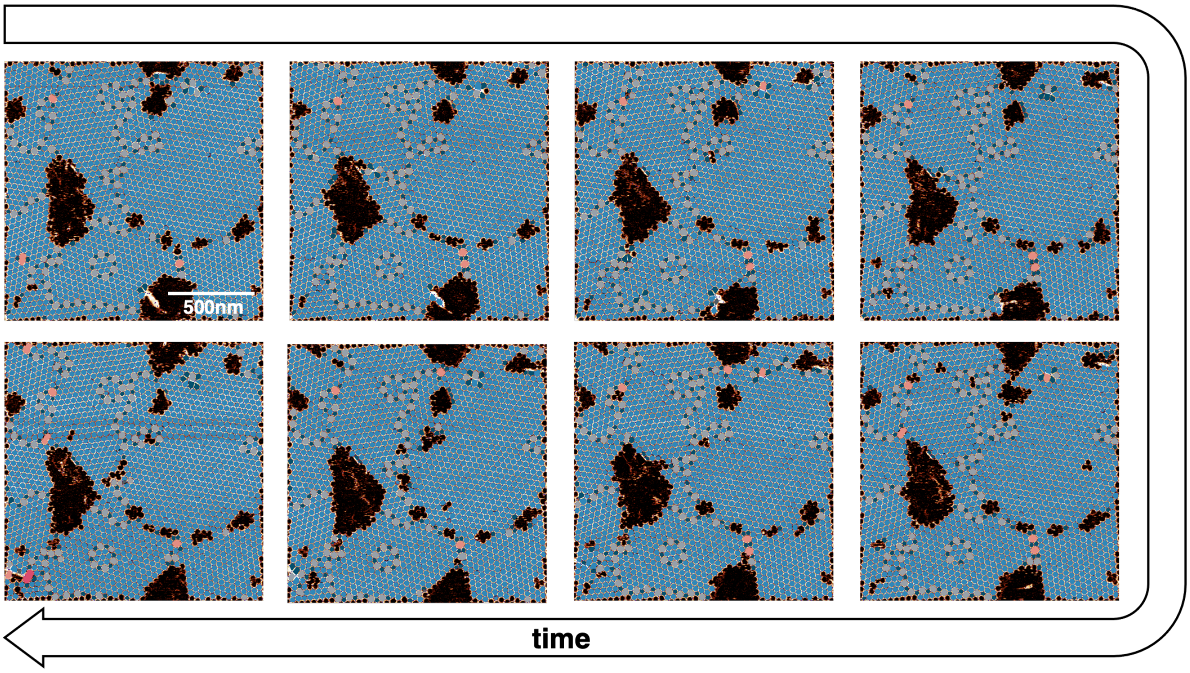

Figure S5: Defects at elevated 3PS concentration.
At increased 3PS concentration, the number of defects observed in a fixed area increases. For this set of images, the first image was acquired after a day of incubation of short 3PS (30nM) on mica in 10mM $MgCl_2$ + 100mM NaCl. Imaging rate was 0.4 frames per minute. We observed a high density of the two types of defects (grain boundary and ring defect) shown in Fig. 3. Additionally, we observed some vacancy defects that were highly dynamic.

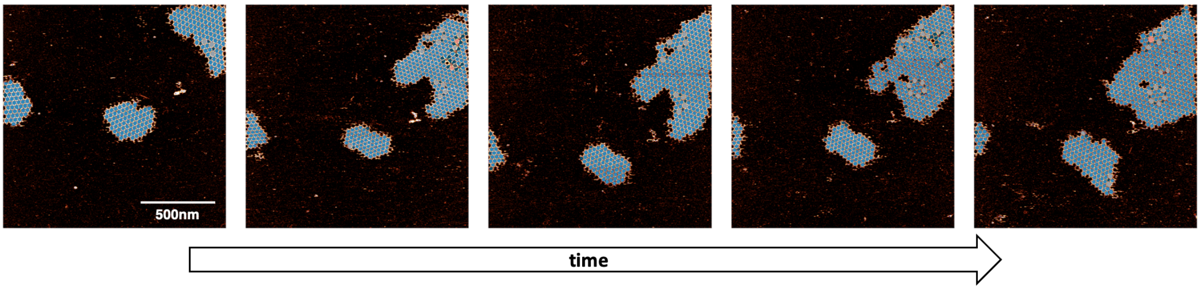

Figure S6: Formation of ring defects.
Here, we observe the formation of a ring defect while islands continue to grow. The defects originated at the island periphery and, as the island grew, the defective region continued to expand, contributing to the formation of observed circular ring patterns. The first image was acquired after 10 minutes incubation of short 3PS (6nM) on mica in 10mM $CaCl_2$. Imaging rate was 0.3 frames per minute.

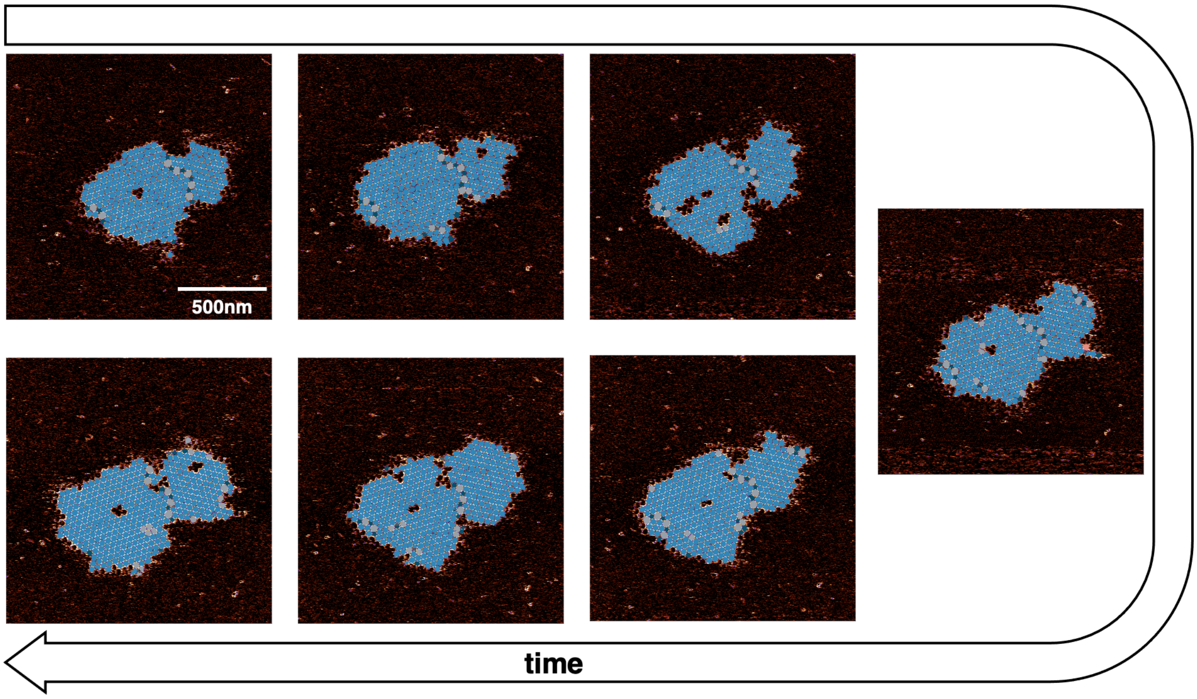

Figure S7: Dynamics of defects and defect correction.
As two coalesced grains, separated by a grain boundary, grow, the grain boundary also extends along the neighboring grains, maintaining its characteristic pentagon-heptagon pattern. This same pattern can emerge on the periphery of an island. As the island continues to grow, this type of defect may result in the formation of a ring defect, as shown in Figure S6. However, the defect can also be corrected, as demonstrated in this case, before the ring pattern is fully formed.
The first image was acquired after 15 minutes incubation of long 3PS with rigid interface on mica in 10mM $MgCl_2$ + 100mM NaCl. Imaging rate was 0.22 frames per minute.

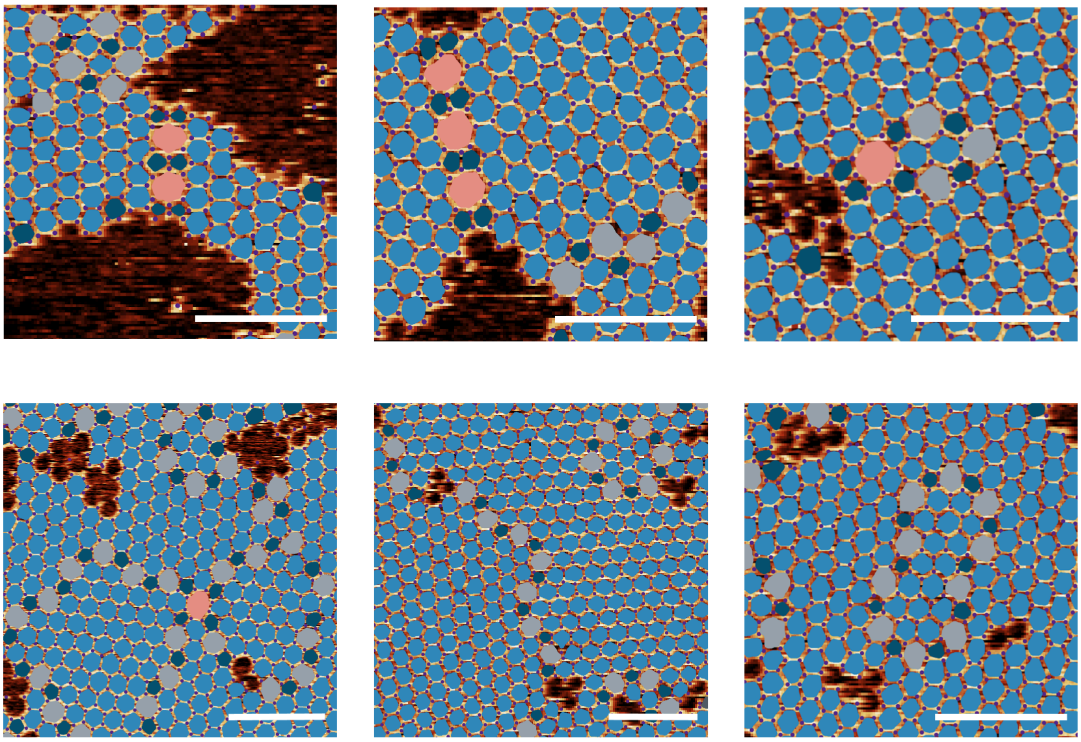

Figure S8: Defect patterns.
The AFM images were acquired after an overnight assembly of short 3PS in 10mM $MgCl_2$ and 100mM NaCl. The octagons are usually flanked by 4 pentagons while the heptagons are flanked by 2 pentagons. The scale bars are 100nm. Pentagons, hexagons, heptagons, and octagons are dark blue, blue, gray, and pink respectively.

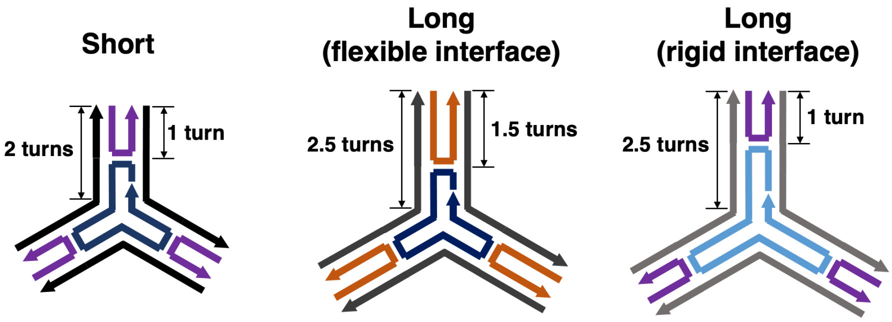

Figure S9: Different 3PS designs used in this study.
In this work, we conducted experiments with the standard blunt-ended 3PS tile (short)[1] as well as two variants with arms that are half a turn longer than the original 3PS[2]. The long 3PS in the middle has not only longer arms compared to the short 3PS but also increased flexibility at the interface due to the extended length beyond the Holliday junction. In contrast, the other long 3PS design (right) maintains the same length after the Holliday junction, making its interface as rigid as the standard 3PS.

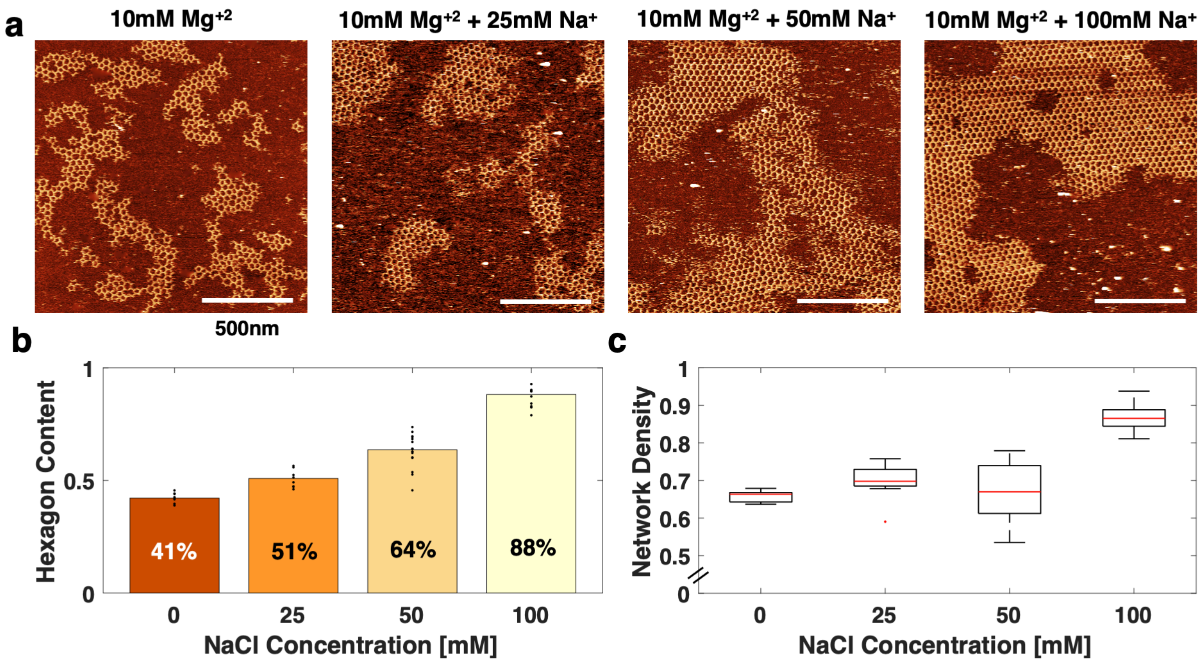

Figure S10: Steady-state networks of long 3PS with rigid interface in the presence of Mg^{2+} and Na^{+}. a, AFM images of the steady state networks in the presence of 10mM $MgCl_2$ and 0mM, 25mM, 50mM, and 100mM NaCl from left to right. **b,** Weighted mean of the hexagon content as a function of the NaCl concentration. **c,** Weighted mean of the Network Density of the resulting networks as a function of the NaCl concentration. Similar to short 3PS, we recorded an increase in both crystallinity and radiality as the Na^{+} concentration increases.

Considering the 3PS with flexible interface is not only more flexible at the interface, but also globally more flexible due to the extended arm length, we conducted experiments with a long 3PS with rigid interface, possessing the same arm length as long 3PS but with the Holliday junction moved half a turn closer to the blunt ends. AFM measurements post-overnight assembly of long 3PS revealed similar radial crystalline lattices, with slightly more defects compared to short and long 3PS due to increased core flexibility, independent of interface flexibility and particle size.

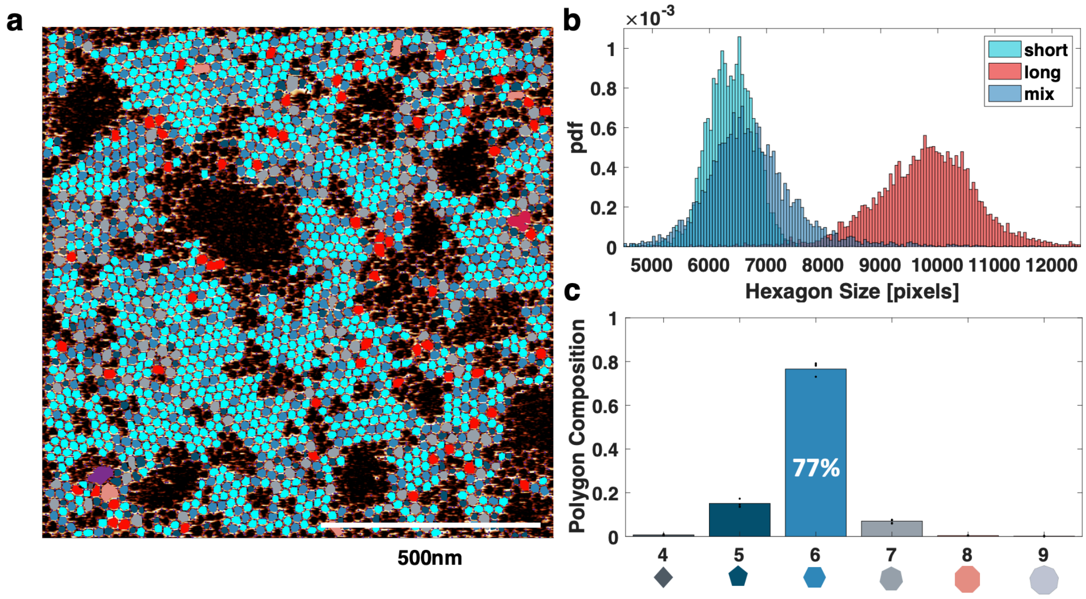

Figure S11: Self-sorting experiment with 2.95nM short 3PS and 0.55nM long (rigid interface) 3PS in the presence of 10mM Mg^{2+} and 100mM Na^{+}. a, An AFM image of the steady state assembly. Hexagons classified as short 3PS only (cyan), long 3PS only (red) and mixture (blue). **b,** Probability density functions (pdf) of hexagon areas for short only (cyan), long rigid only (red) and self-sorting experiment (blue). **c,** The distribution of the polygons formed at the steady-state networks at the self-sorting experiment.

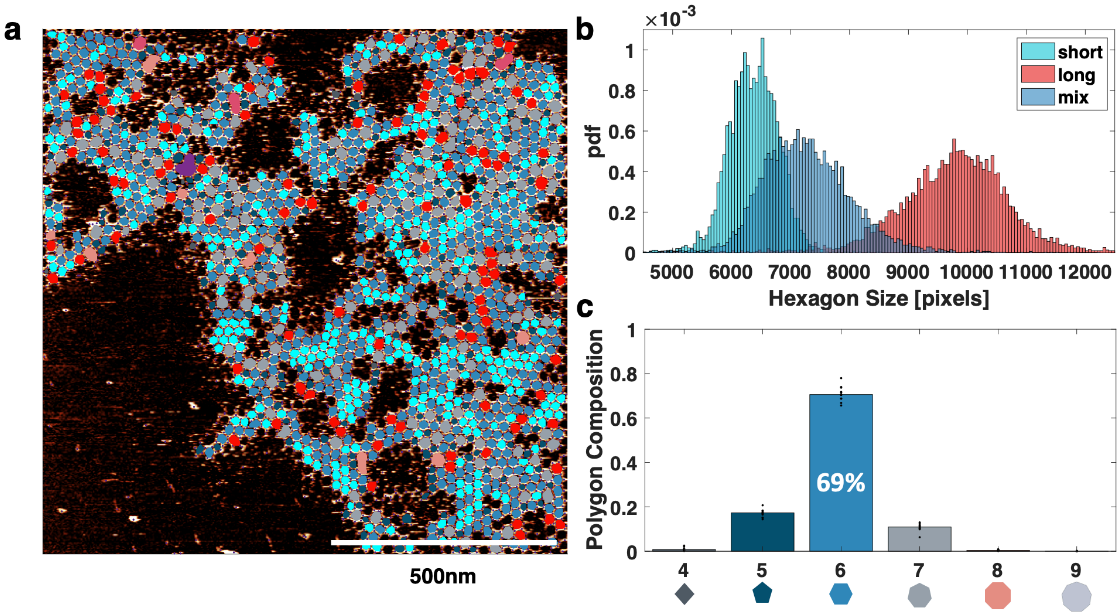

Figure S12: Self-sorting experiment with 3.3nM short 3PS and 1.5nM long (rigid interface) 3PS in the presence of 10mM Mg^{2+} and 100mM Na^{+}. a, An AFM image of the steady state assembly. Hexagons classified as short 3PS only (cyan), long 3PS only (red) and mixture (blue). **b,** Probability density functions (pdf) of hexagon areas for short only (cyan), long rigid only (red) and self-sorting experiment (blue). **c,** The distribution of the polygons formed at the steady-state networks at the self-sorting experiment.

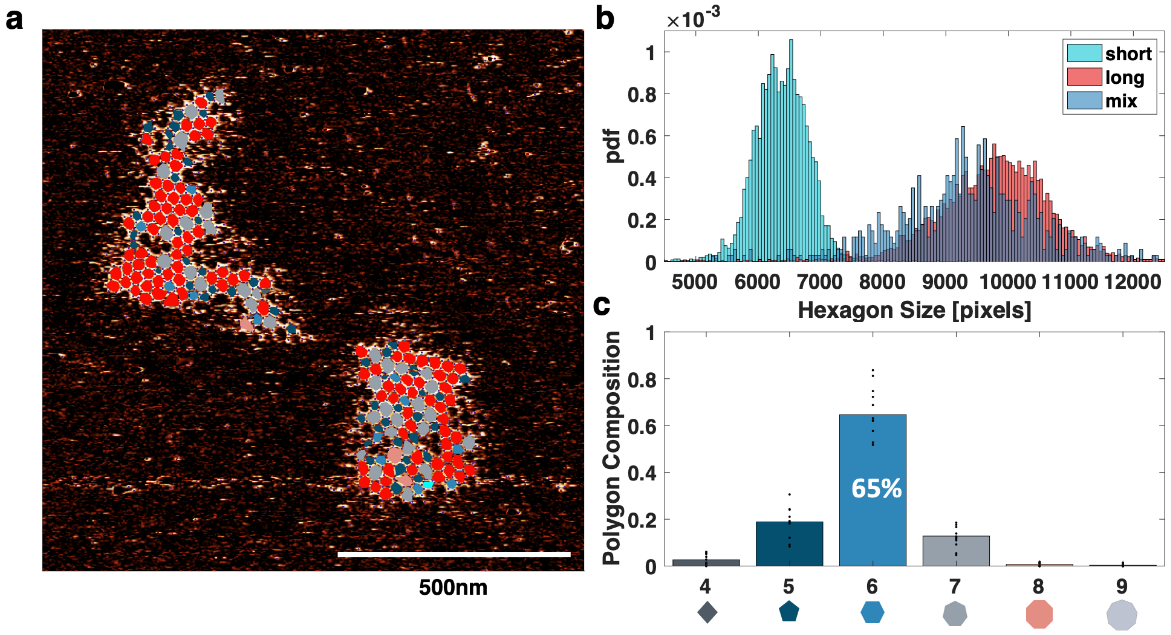

Figure S13: Self-sorting experiment with 1.65nM short 3PS and 1.7nM long (rigid interface) 3PS in the presence of 10mM Mg^{2+} and 100mM Na^{+}. a, An AFM image of the steady state assembly. Hexagons classified as short 3PS only (cyan), long 3PS only (red) and mixture (blue). **b,** Probability density functions (pdf) of hexagon areas for short only (cyan), long rigid only (red) and self-sorting experiment (blue). **c,** The distribution of the polygons formed at the steady-state networks at the self-sorting experiment.

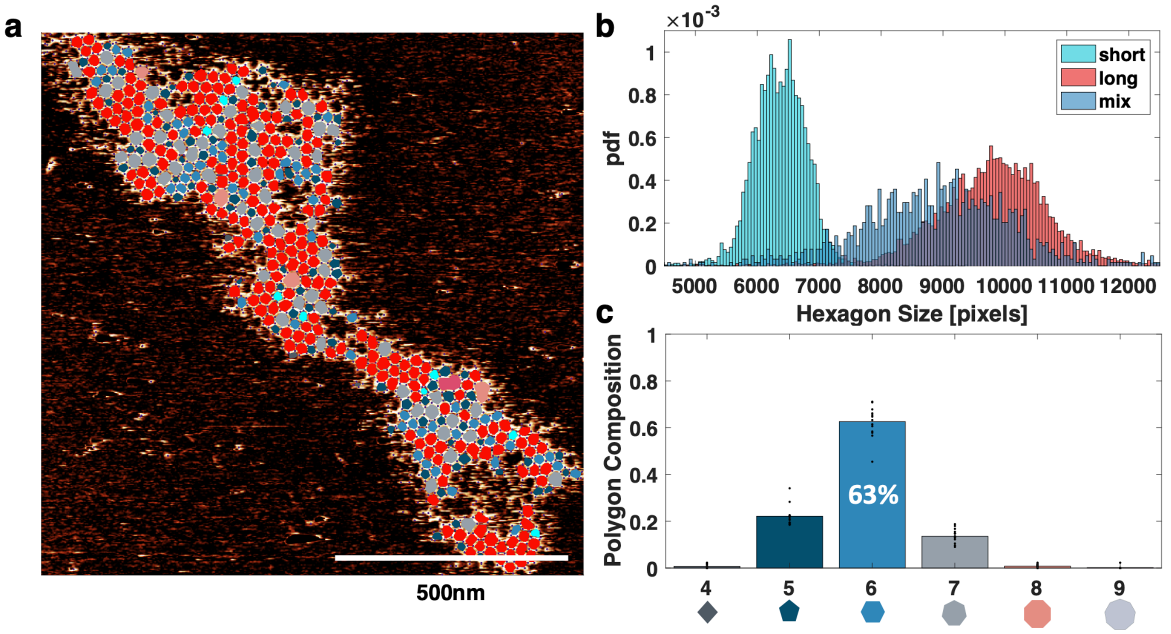

Figure S14: Self-sorting experiment with 2.2nM short 3PS and 1.7nM long (rigid interface) 3PS in the presence of 10mM Mg^{2+} and 100mM Na^{+}. a, An AFM image of the steady state assembly. Hexagons classified as short 3PS only (cyan), long 3PS only (red) and mixture (blue). **b,** Probability density functions (pdf) of hexagon areas for short only (cyan), long rigid only (red) and self-sorting experiment (blue). **c,** The distribution of the polygons formed at the steady-state networks at the self-sorting experiment.

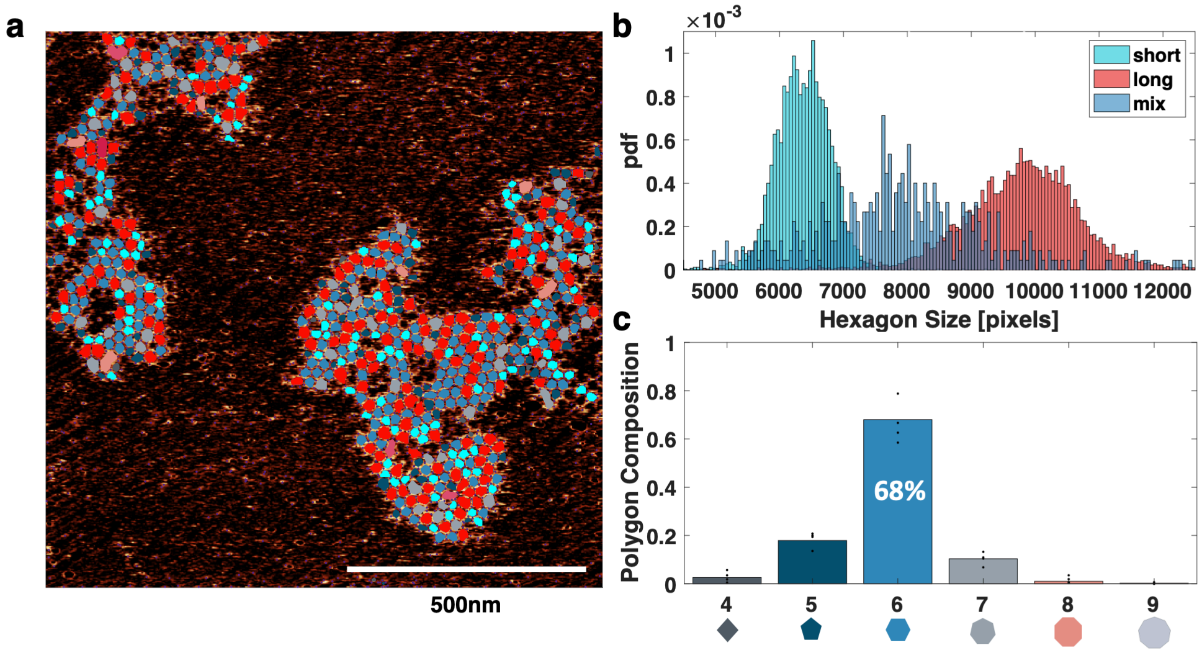

Figure S15: Self-sorting experiment with 3.3nM short 3PS and 1.7nM long (flexible interface) 3PS in the presence of 10mM Mg^{2+} and 100mM Na^{+}. a, An AFM image of the steady state assembly. Hexagons classified as short 3PS only (cyan), long 3PS only (red) and mixture (blue). **b,** Probability density functions (pdf) of hexagon areas for short only (cyan), long rigid only (red) and self-sorting experiment (blue). **c,** The distribution of the polygons formed at the steady-state networks at the self-sorting experiment.

Table S1: The sequences of the DNA strands

**3PS design**	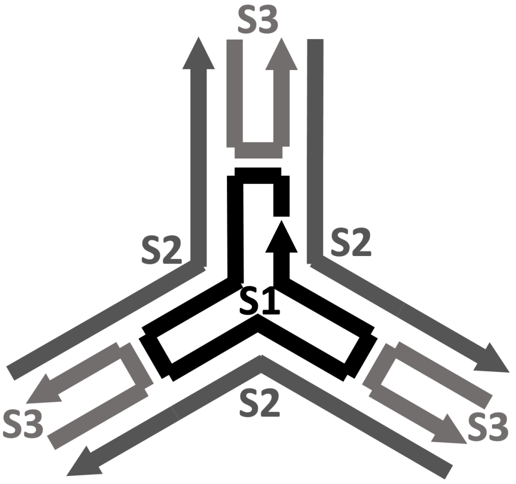
**Short 3PS**	
S1	5’-AGGCACCATCGTAGGTTTCTTGCCAGGCACCATCGT<br>AGGTTTCTTGCCAGGCACCATCGTAGGTTTCTTGCC-3’
S2	5’-ACTATGCAACCTGCCTGGCAAGCCTACGATGGACACGGTAACG-3’
S3	5’-CGTTACCGTGTGGTTGCATAGT-3’
**Long 3PS (flexible interface)**	
S1	5’-AGGCACCATCGTAGGTTTCTTGCCAGGCACCATCGT<br>AGGTTTCTTGCCAGGCACCATCGTAGGTTTCTTGCC-3’
S2	5’-ACTGTAATCGTCAACCTGCCTGGCAAG<br>CCTACGATGGACATTCGGTCTGAACG-3’
S3	5’-CGTTCAGACCGAATGTGGTTGACGATTACAGT-3’
**Long 3PS (rigid interface)**	
S1	5’-AGTAGGCACCAGTCGGATACAGGCTTGACATAGTAGGCACCAGTCG<br>GATACAGGCTTGACATAGTAGGCACCAGTCGGATACAGGCTTGACAT-3’
S2	5’-ACTATGCAACCTGCCTACTATGTCAAGT<br>TTCCTGTATCCGACTGGACACGGTAACG-3’
S3	5’-CGTTACCGTGTGGTTGCATAGT-3’

References

1. He, Y., Chen, Y., Liu, H., Ribbe, A. E. & Mao, C. Self-assembly of hexagonal DNA two-dimensional (2D) arrays. *J Am Chem Soc* **127**, 12202–12203 (2005).
2. Caroprese, V. *et al.* Structural flexibility dominates over binding strength for supramolecular crystallinity. *bioRxiv* (2023) doi:10.1101/2023.09.04.556250.
